# 506. Week 26 Interim Analysis of Implementation and Delivery of Long-Acting Injectable Cabotegravir for PrEP in a Community Pharmacy Setting

**DOI:** 10.1093/ofid/ofae631.158

**Published:** 2025-01-29

**Authors:** Elyse Tung, Alexi Duenas, Peter Shalit

**Affiliations:** Kelley-Ross Pharmacy, Seattle, WA; Kelley-Ross Pharmacy, Seattle, WA

## Abstract

**Background:**

For years, pharmacists have successfully managed oral PrEP therapy through collaborative agreements. The recent availability of long-acting PrEP injectables requires new strategic development for treatment delivery. We aim to demonstrate that a pharmacist-managed PrEP program using long-acting injectable cabotegravir (CAB-LA) in a community pharmacy setting is feasible, as measured by at least 50% of patients retained in care and acceptable to patients.

**Figure 1.**
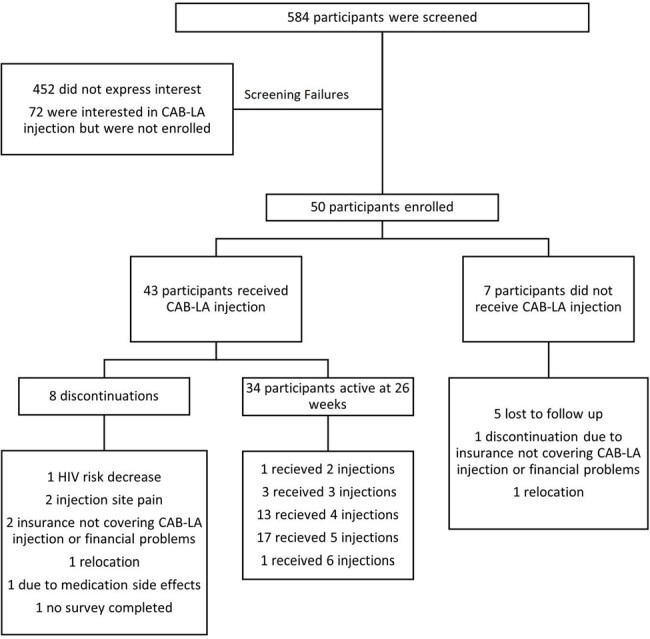
Screening and Follow Up

**Methods:**

This is a 26-week interim analysis of an observational, mixed methods study of 50 participants who have enrolled and been in care at Kelley-Ross Pharmacy in Seattle, Washington from June 1, 2023, to April 26, 2024. The protocol was approved by the institutional review board. Data was collected from both patient surveys and retrospective chart review by study investigators. Participants completed a baseline survey and benefits investigation for CAB-LA for PrEP after enrollment. Follow up surveys were collected at every visit, including early discontinuation. Feasibility outcomes include proportion of participants recruited and retained in PrEP care and adherence (Table 2). Acceptability outcome of patient satisfaction is measured through qualitative data generated from surveys, represented as a Likert scale quantified with variance applied (Figure 2).

**Table 1.**
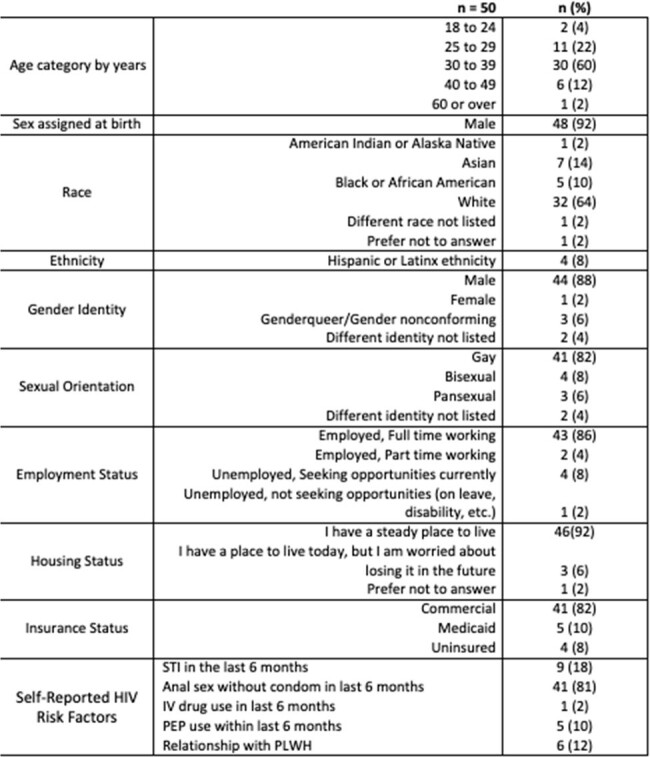
Baseline Characteristics

**Results:**

Fifty participants were enrolled in the study, 43 (86%) of who received an initial injection of CAB-LA. Thirty-five (81%) participants are retained in care through 26 weeks. Out of the 122 injections given, 115 (94%) were on time. Baseline participant satisfaction survey responses are detailed in Figure 2. There were no HIV seroconversions while in the study. Out of the 43 participants who received CAB-LA, eight discontinued prior to week 26 (Figure 1).

**Table 2.**
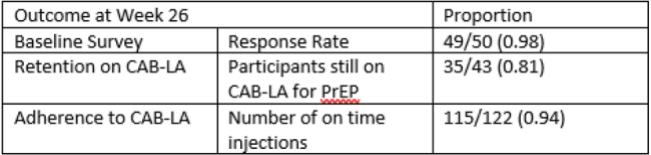
Outcomes

**Conclusion:**

Our data show that a pharmacist-managed CAB-LA for PrEP program in a community pharmacy setting is feasible and acceptable. Patients who were started on CAB-LA for PrEP had a very low discontinuation rate at 26 weeks and expressed a high degree of satisfaction in patient surveys. Reasons for discontinuation are varied among participants. This interim analysis supports the implementation of a pharmacist-managed CAB-LA program in a community pharmacy setting.

**Figure 2.**
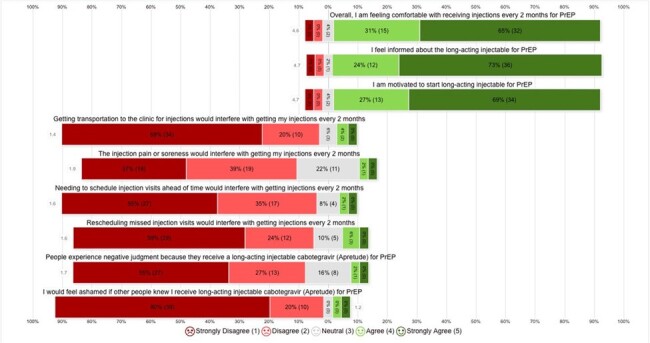
Baseline Survey Participant Barriers and Facilitators of CAB-LA for PrEP

Likert scales quantified with variance applied. The point scale is as follows: 1- strongly disagree, 2- disagree, 3- neutral, 4- agree, 5, strongly agree. Participants completed baseline survey at the time of enrollment, prior to first CAB-LA injection. One participant did not complete the survey and was eliminated from the data set for the above parameters.

**Disclosures:**

**Elyse Tung, PharmD, BCACP**, Gilead Sciences Inc.: Advisor/Consultant|Gilead Sciences Inc.: Honoraria|National HIV PrEP Curriculum: Advisor/Consultant|Viiv Healthcare Ltd.: Advisor/Consultant **Peter Shalit, MD, PhD**, Gilead Sciences: Grant/Research Support|Gilead Sciences: Honoraria|ViiV Healthcare: Advisor/Consultant|ViiV Healthcare: Grant/Research Support|ViiV Healthcare: Honoraria

